# Phenotypic and genomic characterization of the first alkaliphilic aceticlastic methanogens and proposal of a novel genus *Methanocrinis* gen.nov. within the family *Methanotrichaceae*

**DOI:** 10.3389/fmicb.2023.1233691

**Published:** 2023-10-11

**Authors:** Maria A. Khomyakova, Alexander Y. Merkel, Alexander I. Slobodkin, Dimitry Y. Sorokin

**Affiliations:** ^1^Winogradsky Institute of Microbiology, FRC Biotechnology Russian Academy of Sciences, Moscow, Russia; ^2^Department of Biotechnology, Delft University of Technology, Delft, Netherlands

**Keywords:** methanogenesis, soda lake, terrestrial mud volcano, (halo)alkaliphily, *Methanothrix*, *Methanocrinis*

## Abstract

Highly purified cultures of alkaliphilic aceticlastic methanogens were collected for the first time using methanogenic enrichments with acetate from a soda lake and a terrestrial mud volcano. The cells of two strains were non-motile rods forming filaments. The mud volcano strain M04Ac was alkalitolerant, with the pH range for growth from 7.5 to 10.0 (optimum at 9.0), while the soda lake strain Mx was an obligate alkaliphile growing in the pH range 7.7–10.2 (optimum 9.3–9.5) in the presence of optimally 0.2–0.3 M total Na^+^. Genomes of both strains encoded all enzymes required for aceticlastic methanogenesis and different mechanisms of (halo)alkaline adaptations, including ectoine biosynthesis, which is the first evidence for the formation of this osmoprotectant in archaea. According to 16S rRNA gene phylogeny, the strains possessed 98.3–98.9% sequence identity and belonged to the obligately aceticlastic genus *Methanothrix* with *M. harundinaceae* as the most closely related species. However, a more advanced phylogenomic reconstruction based on 122 conserved single-copy archaeal protein-coding marker genes clearly indicated a polyphyletic origin of the species included in the genus *Methanothrix*. We propose to reclassify *Methanothrix harrundinacea* (type strain 8Ac^T^) into a new genus, *Methanocrini*s gen. nov., with the type species *Methanocrinis harrundinaceus* comb. nov. We also propose under SeqCode the complete genome sequences of strain Mx^Ts^ (GCA_029167045.1) and strain M04Ac^Ts^ (GCA_029167205.1) as nomenclatural types of *Methanocrinis natronophilus* sp. nov. and *Methanocrinis alkalitolerans* sp. nov., respectively, which represent other species of the novel genus. This work demonstrates that the low energy aceticlastic methanogenesis may function at extreme conditions present in (halo)alkaline habitats.

## Introduction

Methanogenic archaea use four recognized pathways of methane formation determined by the substrate, namely hydrogenotrophic (utilizing H_2_, formate and CO), methylotrophic (dismutating C_1_-methylated compounds), methyl-reducing (utilizing C_1_-methylated compounds as electron acceptor and H_2_/formate as electron donor), and aceticlastic (utilizing acetate) (Evans et al., 2020; Ferry, 2020; Kurth et al., 2020). Life at high pH and high salt concentrations is energy-costly and is mostly dominated by prokaryotes with a high energy yield of their catabolism (Oren, 1999, 2011). Under alkaline conditions (>pH 9.0), hydrogenotrophic methanogenesis is considered to be the dominant methanogenic pathway (Sorokin et al., 2015; Wormald et al., 2020), and little is known about aceticlastic methanogenesis in alkaline environments. High pH favors the dissociation of acetic acid to its anion (CH_3_COO^−^), preventing transmembrane diffusion (Welte et al., 2014). Thus, under alkaline conditions, the transport of acetate into the cell depends on the acetate transporter, and aceticlastic methanogenesis is likely to be energetically less favorable than hydrogenotrophic methanogenesis (Welte et al., 2014; Wormald et al., 2020).

The acetate-dependent methanogenesis is performed only by two genera–*Methanosarcina* and *Methanothrix* (formerly *Methanosaeta*) (Boone et al., 2001; Akinyemi et al., 2020). *Methanosarcina* species are facultative acetotrophs with a low substrate affinity for acetate; many *Methanosarcina* spp. can also use other pathways, preferring the C_1_-methylated compounds as substrates. A number of *Methanosarcina* species have been isolated from marine habitats (Sowers et al., 1984; von Klein et al., 2002). All *Methanosarcina* species have pH optimum in the range of 6.0–7.5. In contrast, the genus *Methanothrix* includes obligately acetotrophic species with a high affinity for acetate and dominating in freshwater terrestrial habitats (Liu, 2010). Considering a very low energy yield available in obligately acetotrophic members of the genus *Methanothrix*, it is not surprising that, so far, only a single salt-tolerant species–“*Methanosaeta pelagica*”–has been found in a marine habitat (Mori et al., 2012). Recently, however, an obligate acetotrophy of *Methanothrix* was questioned by the discovery of the ability to utilize electrons to activate the otherwise inert CO_2_-reducing branch encoded in the genome, either in syntrophy with *Geobacter* by a direct electron transfer mechanism or in the presence of electron-conducting minerals (Rotaru et al., 2014; Zhao et al., 2018). Conversion of acetate to methane at alkaline conditions has never been demonstrated for the family *Methanotrichaceae* by cultivation approach, although *Methanothrix*-related sequences were found in the low-salt alkaline lake (Antony et al., 2012).

Organisms dominating microbial communities in saline soda lakes (at least those that were cultured) are mostly obligate haloalkaliphiles, growing optimally in Na-carbonate brines of variable concentrations with pHs above nine. The cultured haloalkaliphilic methanogens from the Kulunda Steppe soda lakes (Altai region, Russia) are represented by the methylotrophic genera *Methanosalsum* and *Methanolobus*, while the dominant hydrogenotrophic genus *Methanocalculus* is involved in syntrophic oxidation of acetate up to extremely high salinities of 3 M total Na^+^, unprecedented in the neutral salt habitats (Sorokin et al., 2015, 2016).

Terrestrial mud volcanoes (TMVs) are important natural sources of methane emission. These geological structures located on the Earth's surface are connected to deep and pressurized hydrocarbon reservoirs. TMVs may provide a direct means to recover microbial communities inhabiting subsurface fluids and sediments through fracture networks, often extending to a depth of several kilometers (Mazzini and Etiope, 2017). Although most hydrocarbon gases released from TMVs are thermogenic, sometimes emission of biogenic methane can reach 20% of the total methane (Etiope et al., 2009). The archaeal part of the TMVs microbial communities usually contains many anaerobic methane-oxidizing archaea (ANME) and a small number of methanogens (Alain et al., 2006; Niederberger et al., 2010; Merkel et al., 2021; Tu et al., 2022). Mud volcanic fluids of the Kerch–Taman region often have high pH values due to a presence of sodium carbonates (Lavrushin et al., 2021). To date, neither methanogens nor any *Archaea* from TMVs were obtained in a pure or highly purified culture and physiologically characterized.

Here we describe properties of two obligately acetotrophic methanogenic strains from alkaline habitats–saline soda lake and terrestrial mud volcano–representing first examples of alkaliphilic aceticlastic methanogens. Together with *Methanothrix harundinacea*, they form a distinct new, genus-level lineage in the family *Methanothrichaceae*, for which we propose the name *Methanocrinis*.

## Materials and methods

### Sampling site and cultivation conditions

Aceticlastic strain Mx was enriched from a sample of the top 5–10 cm deep anaerobic (sulfidic) sediment overlaid by the bottom brines taken from the soda lake Tanatar-6 in Kulunda Steppe (Altai, Russia; N51°37′/E79°48′) in July 2011. The total salinity, carbonate alkalinity, and pH of the brines were 160 g l^−1^, 1.7 M and 10.04, respectively (Sorokin et al., 2015). In the laboratory, the 20 ml of the sediment:brine slurry (1:5, v/v) was homogenized by shaking, and the coarse sandy sediment fraction was removed by a series of low-speed centrifugations. The remaining fine sediment particles were further collected by a high-speed centrifugation, and the pellet was resuspended in 2 ml anaerobic culture medium (see below) and used as an inoculum for the enrichment cultivation. A sodium carbonate/bicarbonate-based buffer with pH 9.5 containing 0.5 M of total Na^+^ as carbonates, 0.1 M NaCl, and 1 g l^−1^ K_2_HPO_4_ was used as a mineral base medium for the enrichment. After sterilization, the medium was supplemented with 4 mM NH_4_Cl, 1 mM MgCl_2_, acidic trace metals (1 ml l^−1^) and vitamins (1 ml l^−1^) (Pfennig and Lippert, 1966), basic Se/W solution (1 ml l^−1^) (Plugge, 2005), and yeast extract (0.01 g l^−1^). Sodium acetate was used as a substrate for methanogenesis at concentrations of 20–100 mM (added from a 2 M sterile stock solution). Eighty ml medium was dispensed into 115 ml sterile serum bottles, supplemented with 0.5 mM Na_2_S (from 1 M filter-sterilized solution), and oxygen was removed by three cycles of evacuation and flushing with sterile argon gas. Final reduction of the medium was achieved by adding a drop of 10% dithionite solution in 1 M NaHCO_3_. The primary enrichments (three parallel incubations) were inoculated with 1 ml of the fine sediment slurry (see above) and incubated at 28°C statically with periodic measurements of methane concentrations in the gas phase. When the methane concentration reached 30–40% in the gas phase and visible turbidity appeared in the undisturbed liquid phase, the sediment-free cultures were transferred twice 1:100 under the same conditions, and the last successful transfer was used to inoculate a 1:10 dilution series under the same conditions in 15 ml Hungate tubes with 10 ml medium. The dilution series were repeated multiple times in combination with a preliminary low-speed centrifugation step, which allowed enrichment for filamentous *Methanothrix*-like cells. Numerous attempts have also been made to obtain colonial growth on plates with agar-shake techniques incubated in 2.5 l anaerobic jars (Oxoid) under argon gas phase and in the presence of an O_2_-scavenging catalyzer, but they were not successful.

Strain M04Ac was isolated from a sample of mud collected from the active griffin of terrestrial mud volcano Gnilaya Gora, Taman Peninsula, Krasnodar region, Russia. Coordinates of the sampling point were 45.251°N, 37.436°E. Samples were collected in May 2017, from the upper 20 cm of mud, pH 8.5, temperature 21°C, 15.7 mM Cl^−^, 5.3 mM SO${\;}_{4}^{2-}$. Samples were taken anoxically in tightly stoppered plastic bottles and transported to the laboratory. Enrichment and isolation were performed in the liquid medium of the following composition (per liter distilled water): KH_2_PO_4_, 0.33 g; NH_4_Cl, 0.33 g; KCl, 0.33 g; MgCl_2_·6H_2_O, 0.33 g; CaCl_2_·6H_2_O, 0.033 g; NaCl, 10.00 g; NaHCO_3_, 2.00 g; Na_2_S·9H_2_O, 0.5 g; 1 ml trace element solution (Slobodkin et al., 2012), 1 ml vitamin solution (Wolin et al., 1963), and 1 ml resazurin solution (0.001g l^−1^). The medium was prepared by boiling and cooling it under N_2_ (100%) flow. Afterwards, NaHCO_3_, vitamins and Na_2_S·9H_2_O were added. The medium was dispensed in 10 ml aliquots into 17 ml Hungate tubes, and the head space was filled with 100% N_2_ (high-purity grade). The tubes were autoclaved at 1 atm, 121°C for 20 min. The pH of sterile medium was adjusted to 8.0–8.5 at 25°C with 10% sterile anaerobic NaOH solution using anaerobic technique. Sodium acetate from sterile anoxic stock solution was added before inoculation as the growth substrate to a final concentration of 20 mM. Growth of the strains during isolation process was monitored by a phase-contrast microscope (Olympus CX-43 and Zeiss Axioplan Imaging 2 microscope).

### Phenotypic characterization of the strains

Growth of both cultures during physiological experiments was estimated by OD_600_ after vigorous homogenization. To determine cell morphology of the strain M04Ac, transmission electron microscopy was performed using JEM-1400 electron microscope (JEOL, Japan).

All the cultivation experiments were done in triplicate. Temperature (from 10 to 60°C) and pH growth ranges (from 5.0 to 11.0) of the strain M04Ac were determined using the same medium as for the strain purification. For pH experiments, the following Good's buffers (Sigma-Aldrich) were used (30 g l^−1^): MES (pH 6 and 6.5), HEPES (pH 7 and 7.5), Tricine (pH 8.0 and 8.5), CAPSO (pH 9.0 and 9.5), and CAPS (pH 10–11). The NaCl requirement for growth was determined at optimal pH in the medium of the same mineral composition but lacking NaCl. Varying amounts of NaCl (0–10.0%, w/v) were added directly into 50-ml serum bottles before sterilization.

The salinity profile of the strain Mx was studied by using sodium carbonate/bicarbonate buffer with pH 9.5 and a total Na^+^ concentration ranging from 0 to 2 M. The pH range was determined by using HEPES/NaHCO_3_ buffer for the pH range from 6 to 8 and a NaHCO_3_/Na_2_CO_3_ buffer for the range from pH 8.5–11. Both profiles were obtained in growing cultures and in washed (resting) cells, harvested from 0.5 l culture grown at optimal pH–Na^+^ conditions at the end of exponential growth phase. It has to be stressed that the final pH values deviated substantially from the initial values, especially at both extremes and, therefore, the final pHs were measured and are indicated in the results. All growth and cell incubation experiments were done in triplicate.

Methane was analyzed by GC using Chromateck Crystall 5000 (Ufa, Russia); column Hayesep 80–100 mesh, 2 m x 3 mm at 40°C; FID detector at 200°C; and argon as the carrier gas at 25 ml min^−1^. Concentrations of acetate were determined in filtered supernatants after neutralization with 1 M HCl to pH 7. The HPLC conditions were as follows: Animex HPX-87H column at 60°C, eluent 5 mM H_2_SO_4_ at 0.6 ml min^−1^, and UV and RI detectors.

Ectoine was extracted from freeze-dried cells of strain Mx with 80% ethanol enforced by bead beating, followed by centrifugation and filtration through a 0.22 μm filter. The concentration of ectoine was measured with a Shimadzu Prominence-i LC-2030C plus HPLC (Shimadzu, Japan) equipped with a UV detector at 210 nm and a Polaris, NH2, 180 Å, 3 μm, 4.6 × 150 mm column (Agilent Technologies, USA) with a mobile phase of acetonitrile/H_2_O 75/25 (%) at a flow rate of 0.6 ml min^−1^. Oven temperature was set at 30°C. Ectoine and hydroxyectoine standards (Sigma Aldrich, Germany) were used for quantification.

### 16s rRNA gene and genome sequencing and analysis

DNA from mud samples as well as from enrichment cultures was isolated using FastDNA Spin Kit for Soil according to the manufacturer's protocol (MP Biomedicals, Santa Ana, California, USA). Whole-genome sequencing and metagenomic bioinformatics analysis were used to assess the purity of cultures at the final stages of their purification according to methods described in Slobodkin et al. (2023). 16S rRNA gene sequencing was performed as described previously (Slobodkina et al., 2022). The GenBank accession numbers for 16S rRNA gene sequences of strains Mx and M04Ac were KP205578 and OQ918309, respectively. The 16S rRNA gene sequences of the strains were compared with other sequences in GenBank (Benson et al., 1999) by using the BLAST program (Altschul et al., 1990) and by means of the EzBioCloud server (Yoon et al., 2017) (http://www.ezbiocloud.net) to identify their closest relatives. Sequences were aligned by MAFFT v7.427 (G-INS-i strategy) (Nakamura et al., 2018) for 16S rRNA gene-based phylogenetic analyses. A shotgun WGS library preparation and sequencing for both strains were done in BioSpark Ltd., Moscow, Russia using KAPA HyperPlus Library Preparation Kit (KAPA Biosystems, UK) according to the manufacturer's protocol and NovaSeq 6000 system (Illumina, San Diego, CA, USA) with the reagent kit, which can read 100 nucleotides from each end. Raw reads were processed with Trimmomatic (Bolger et al., 2014) for adapter removal and quality filtering. Assembly of reads was performed by Unicycler v0.4.8 (Wick et al., 2017). These Whole Genome Shotgun projects have been deposited at DDBJ/ENA/GenBank under the accession JARFPL000000000 for M04Ac and JARFPK000000000 for Mx. For the satellite bacteria, Whole Genome Shotgun projects have been deposited at DDBJ/ENA/GenBank under the BioProject ID PRJNA1010173. Metagenomic bioinformatics approaches were used to separate genomes in binary cultures as described in Slobodkin et al. (2023). Metawrap v1.3 (Uritskiy et al., 2018) quant bins workflow was used to determine the relative abundance of the microorganisms in binary cultures by analyzing coverage using Salmon (Patro et al., 2017). Gene search and annotation were performed by the Prokaryotic Genome Annotation Pipeline (PGAP) (Tatusova et al., 2016).

For phylogenetic reconstructions, 122 conserved single-copy archaeal protein-coding marker genes (Parks et al., 2020) as well as 16S rRNA gene were used. The trees were built using the IQ-TREE 2 program (Minh et al., 2020) with fast model selection via ModelFinder (Kalyaanamoorthy et al., 2017) and ultrafast approximation for phylogenetic bootstrap (Hoang et al., 2018), as well as approximate likelihood-ratio test for branches (Anisimova and Gascuel, 2006). The average amino acid identity (AAI) between the selected genomes was calculated using the EzAAI v1.1 (Kim et al., 2021). The average nucleotide identity (ANI) between the selected genomes was calculated using the pyani v0.2.12 (Pritchard et al., 2016). *in silico* DDH were calculated using the Genome-to-Genome distance calculator (GGDC) (Meier-Kolthoff et al., 2013). The relative evolutionary divergence (RED) values were calculated using GTDB-Tk v2 (Chaumeil et al., 2022).

## Results

### Enrichment of the strain Mx

Only a single culture out of multiple attempts whereby acetate served as substrate for methanogenesis in soda lake sediments resulted in an enrichment of an aceticlastic methanogen, while in all other cases acetate was consumed by syntrophic associations of the acetate-fermenting *Ca*. Syntrophonatronum acetioxidans partnered with hydrogenotrophic *Methanocalculus alkaliphilus* (at moderate salinities) or *Mc. natronophilus* (at salinities above 2 M Na^+^) (Sorokin et al., 2014, 2015, 2016). In contrast to the syntrophic pathway dependent on the bacterial partner, in a single positive culture (at 0.6 M total Na^+^ and pH 9.5) enriched from the soda lake Tanatar-6 in Kulunda Steppe, acetate-dependent methanogenesis was not inhibited by the addition of streptomycin and kanamycin, at 0.1 g l^−1^ each. In fact, antibiotics helped to stabilize and increase activity of the aceticlastic organism, whose morphology was very similar to that of *Methanothrix* with a domination of multicellular filaments. Several rounds of dilution to extinction resulted in a highly enriched culture, but it still contained a low number of small bacterial rod-shaped cells apparently resistant to the used antibiotics. Using whole-genome sequencing and metagenomic bioinformatics analysis, we assessed the phylogenetic affiliation and relative abundance of these bacteria. The culture was contaminated with only one type of bacteria, which belonged to the *Bacteroidales* and was represented by only 1% of the community microorganisms. An analysis of the phylogenetic position of this bacterium based on 120 conserved single-copy bacterial protein-coding marker genes is shown in Supplementary Figure 1A. According to GTDB, it belongs to family-level lineage UBA7960 and to the genus-level lineage SKTA01 that consists only of MAGs sequenced from Kulunda hypersaline soda lake sediments. There were no other contaminating microorganisms in the culture. Preincubation with rifampicin (0.1 g l^−1^) for 2–3 days with further dilution series in the absence of antibiotics eliminated the bacterial contamination, but the resulting pure culture was not transferrable and, hence, could not be deposited in a culture collection. The strain was designated as Mx.

### Enrichment of the strain M04Ac

An enrichment culture was initiated by 10% (w/v) inoculation of a mud sample into anaerobic sterile medium with 3,4-dimethoxybenzoic acid (10 mM), which after 3 months of cultivation was replaced by acetate (20 mM) as a substrate and ampicillin (0.5 g l^−1^) for suppression of bacterial growth. After a month of incubation on acetate at 30°C in the dark, the growth of long, non-motile filaments and production of methane was observed. After three subsequent 10% (v/v) transfers, serial 10-fold dilutions in the same liquid medium with the addition of yeast extract (0.05 g l^−1^) significantly enhanced the growth of the culture. The dilution series were repeated several times in combination with a preliminary low-speed centrifugation step (at 2 500 rpm for 7 min) which enriched pellet in filamentous *Methanothrix*-like cells. Incubation with rifampicin (0.1 g l^−1^) for 2–3 days with further dilution series was ineffective as significant growth inhibition of the target organism occurred. Attempts to obtain separate colonies were also unsuccessful either with 1% Gelrite gellan gum or with 1.5% agar as the solidifying agent. When isolating *Methanothrix*-like cells, the satellite bacterium belonging to the *Acholeplasmataceae* family (Supplementary Figure 1B) was isolated, whole-genome sequenced, and tested for antibiotic sensitivity. Further use of a mixture of lincomycin and streptomycin (0.05 g l^−1^ each) resulted in a highly enriched culture. Still, by using whole-genome sequencing and metagenomic bioinformatics analysis, we detected the same contamination represented by *Acholeplasmataceae* bacterium isolated in the previous step; its relative abundance was 2.5%. There were no other contaminating microorganisms in the culture. The resultant highly enriched culture was used for subsequent phenotypic characterization, since further attempts to separate *Methanothrix-*like cells from the bacterial component led to a sharp decrease in the growth rate and, ultimately, to the death of the culture, similar to the purified Mx culture.

### Phylogenetic and environmental distribution analysis

The 16S rRNA gene sequence identity between two novel strains was 98.03%; between *M. harundinacea* 6Ac and strains M04Ac and Mx, it was 98.30 and 98.64%, respectively. The deduced amino acid sequence identities of *mcrA*, the gene encoding the α-subunit of methyl-coenzyme M reductase, between *M. harundinacea* 6Ac and two novel strains were 88.73% for the strain Mx and 88.37% for the strain M04Ac, while between two strains it corresponded to 95.53%. Classification of *M. harundinacea*, M04Ac and Mx strains into three separate species was strongly supported by ANI values (87.30% between two new strains; 85.65% between *M. harundinacea* 6Ac and strain Mx; 85.70% between *M. harundinacea* 6Ac and strain M04Ac). *In silico* DDH estimation between two new strains was 29.4%, between *M. harundinacea* 6Ac and strain Mx−25.0%, and between *M. harundinacea* 6Ac and strain M04Ac−25.5%. AAI value between two new strains was 83.72%, between *M. harundinacea* 6Ac and strain Mx−79.21%, and between *M. harundinacea* 6Ac and strain M04Ac−80.18%. Thus, considering all these parameters, the three studied strains are different species of the same genus. The similarity values of strains M04Ac and Mx to the three validly described representatives of the family *Methanotrichaceae*, based on their 16S rRNA and *mcrA* gene sequences, as well as ANI and AAI analysis, are summarized in Supplementary Table 1.

**Figure 1:**
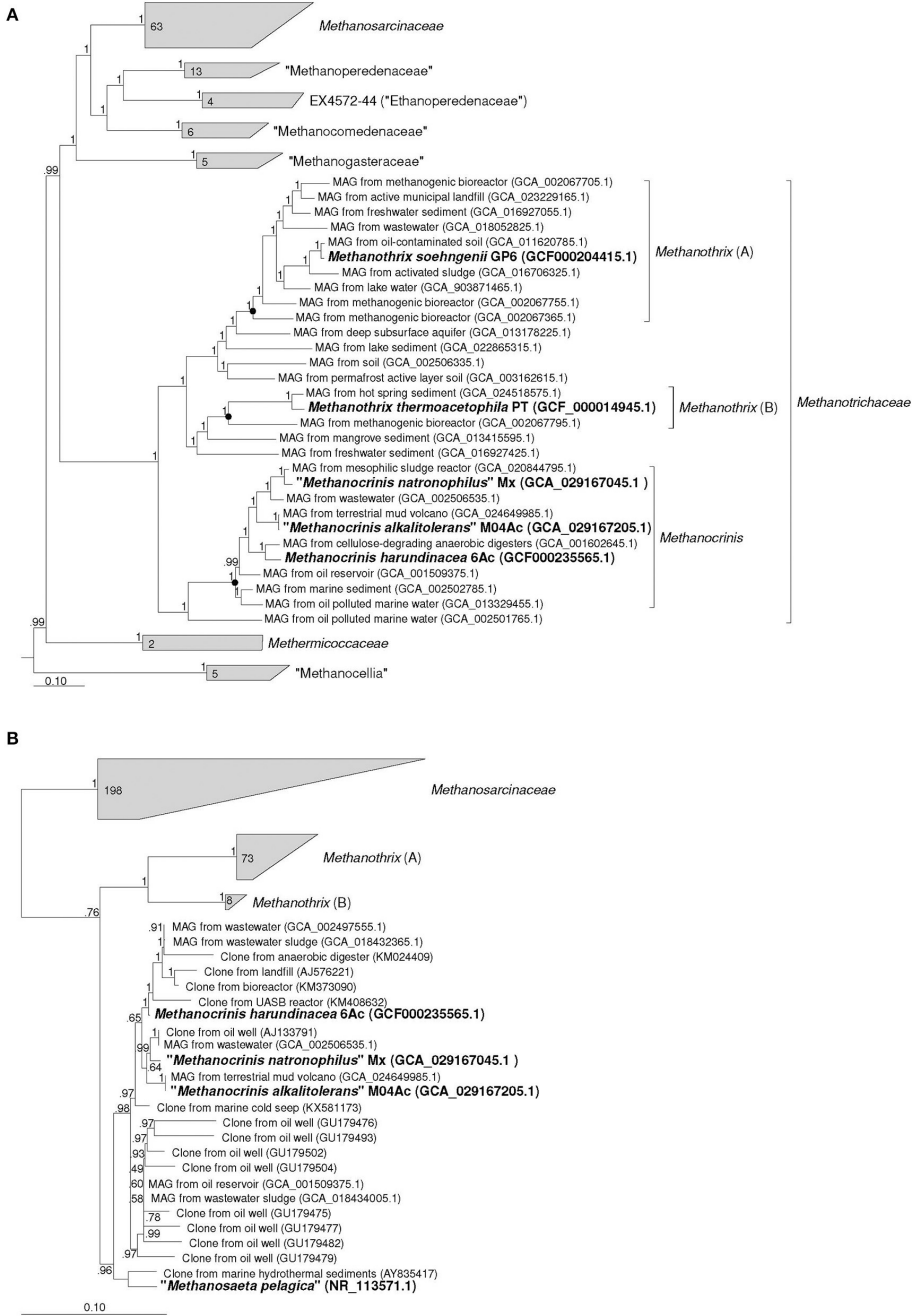
Phylogenomic placement of strains Mx and M04Ac based on **(A)** concatenated amino acid sequences of 122 archaeal single copy conserved marker genes; **(B)** on 16S rRNA gene sequences. The trees were built using the IQ-TREE 2 program (Minh et al., 2020) with fast model selection via ModelFinder (Kalyaanamoorthy et al., 2017) and ultrafast bootstrap approximation (Hoang et al., 2018) as well as approximate likelihood-ratio test for branches (Anisimova and Gascuel, 2006). Bootstrap consensus tree is shown with values placed at the nodes. Bar, 0.1 changes per position.

According to the results of our phylogenomic analysis based on 122 conserved single-copy archaeal protein-coding marker genes (Figure 1A), strains M04Ac and Mx clustered together with *Methanothrix harundinacea* and a number of other metagenome-assembled genomes (MAGs) to form a separate lineage, which was significantly separated from the clusters formed by *Methanothrix soehngenii* and *Methanothrix thermoacetophila*. This separate genus-level group is proposed here to form a new genus, *Methanocrinis* (see below). Our data correlate well with the Genome Taxonomy Data Base (GTDB, Parks et al., 2022), where each validly described *Methanothrix* species forms its own genus-level lineage. In fact, the relative evolutionary divergence (RED) values for these three genera as they are shown in the Figure 1A are 0.872, 0.840, and 0.897 for *Methanothrix* (A), *Methanothrix* (B), and *Methanocrinis*, respectively, whereas, the median value of RED for genus delineation in the GTDB 214 is 0.909. Based on the phylogenomic tree, it is also possible to analyze the origin of the MAGs belonging to these three lineages. While the ecotopes inhabited by all three genera are quite diverse, only *Methanocrinis* group contains MAGs from marine ecotopes, which supports our assumption that the genus *Methanocrinis* contains salt-tolerant species.

The identity values between the 16S rRNA gene sequences of the two new strains and *M*. *harundinacea* on the one hand and *M*. *soehngenii* and *M*. *thermoacetophila* on the other hand strongly confirm that these are different genera of microorganisms. These values fall in the range of 91.54–92.55%. Phylogenetic analysis based on the 16S rRNA gene also confirms the conclusion that each of the currently validly described *Methanothrix* species represents a separate branch at a relatively deep level and that the branch represented by *M*. *harundinacea* is most distantly related (Figure 1B). However, the phylogenetic status of the “*Methanosaeta pelagica*,” which was isolated from a marine habitat (Mori et al., 2012), remains unresolved. This microorganism undoubtedly gravitates toward the proposed genus *Methanocrinis*, but its genome sequence is necessary to resolve its affiliation with this genus.

By outlining the membership of the genus *Methanocrinis* based on the 16S rRNA gene (Figure 1B), we were able to investigate its environmental distribution based on the data available in public databases. Out of 39,243 sequences that belong to *Methanosaetaceae* in the SILVA SSU 138.1 database, we selected 5,276 sequences that belonged to the genus *Methanocrinis* using the BLASTn program. To avoid counting replicates, sequences with a unique combination of “isolation source” and “work title” entries were selected. Thus, a representative selection of 228 16S rRNA gene sequences was obtained (Supplementary Table 2). Among them, sequences found in organic-rich, anthropogenic ecotopes were most common. These include ecotopes representing various kinds of bioreactors (33.5%) and waste disposal systems (9.7%). The second most frequent ecotopes were associated with hydrocarbons, e.g., oil fields and natural gas fields (25.7%). Freshwater ecotopes accounted for 8.7%, coastal ecotopes, such as estuary and mangroves, accounted for 7.3%, and seawater ecotopes accounted for 4.4%. Sequences from various subterranean ecotopes, such as mines and groundwaters, accounted for 5.8%, whereas sequences from mud volcanos accounted for 3.4%. All other types of ecotopes were encountered in single cases.

### Phenotypic characterization of novel aceticlastic strains

**Figure 2:**
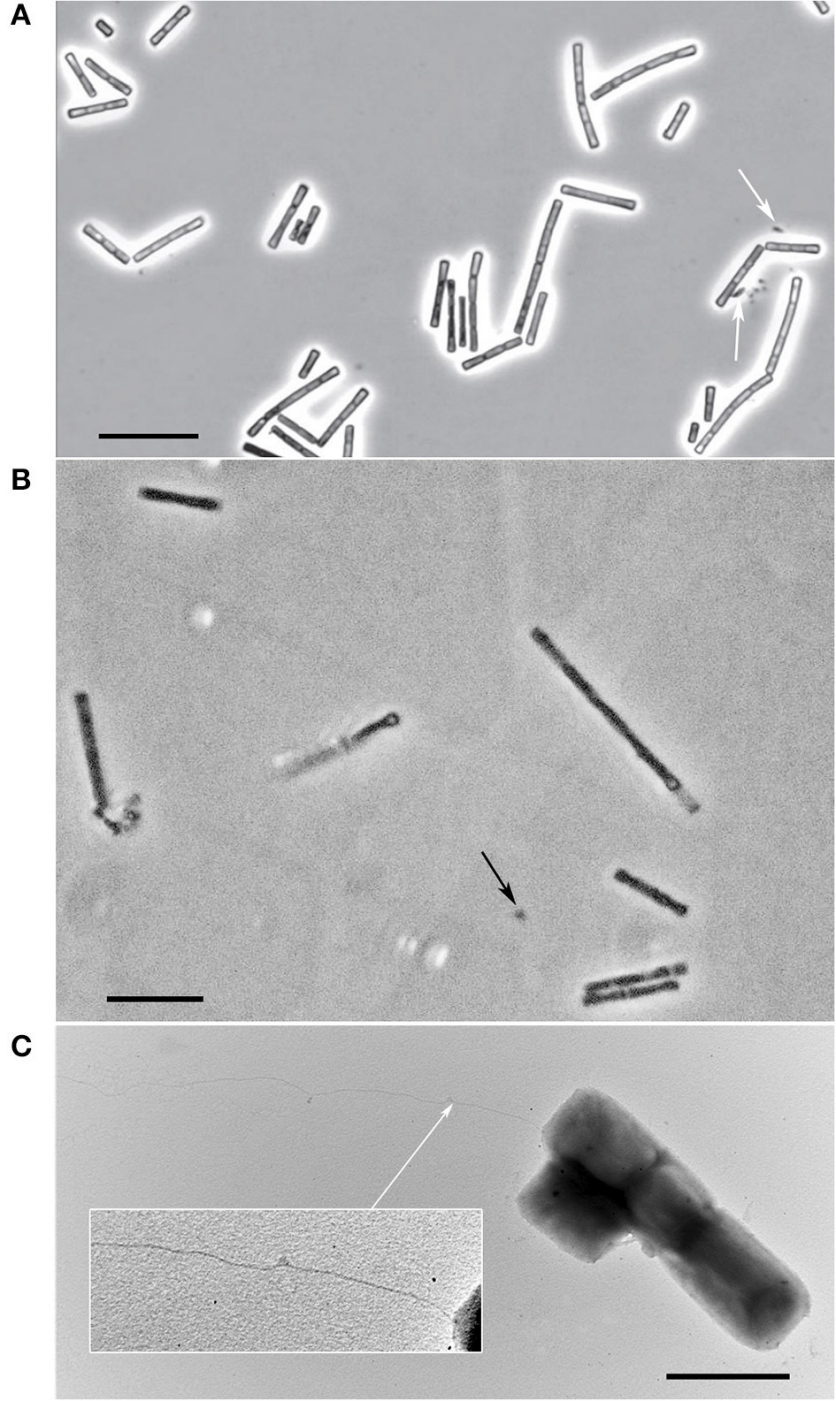
Cellular morphology of strains. **(A)** Phase-contrast photograph of strain Mx. The satellite of the strain is marked with white arrow. Bar, 10 μm. **(B)** Phase-contrast photograph of strain M04Ac. The satellite is marked with black arrow. Bar, 10 μm. **(C)** Electron micrograph of M04Ac cells negatively stained with phosphotungstic acid showing an overall cell morphology and localization of an unknown surface long appendage (marked by white arrow). Bar, 2 μm.

The individual cells of both strains were straight rods with flat ends with cell sizes 1.7–6.5 x 0.9–1.5 and 1.9–4.8 x 0.6–1.0 μm for strain M04Ac and Mx, respectively. They also formed flexible chains of cells within sheaths of variable lengths (Figure 2). The satellite of strain Mx belonging to the *Bacteroidales* group was represented by small sporadic rods (Figure 2A). The satellite culture from *Acholeplasmataceae* family, associated with the strain M04Ac, had a size of about 0.4 μm and was represented by small cocci that were poorly distinguishable in a phase-contrast microscope (Figure 2B). The cells of novel strains were non-motile. However, on electron micrographs of M04Ac cells, long, thin surface appendages of an unknown nature were noticed (Figure 2C). Such fimbria-like structures have never been reported before in any members of the *Methanotrichaceae* family.

Both strains appeared to be typical obligately aceticlastic methanogens similar to other known *Methanothrix* species. From the tested classical methanogenic substrates including H_2_, formate, CO, MeOH, and acetate, only the latter supported growth and methane formation. The resting cells grown with acetate also formed methane only from acetate. For the strain Mx, addition of yeast extract at low concentrations (0.01–0.02 g l^−1^) slightly decreased the usually long lag phase after fresh transfer, but there was no obligate dependence on it. In contrast, low concentrations of yeast extract (0.05 g l^−1^) highly stimulated growth of strain M04Ac, but the percentage of the bacterial component also increased significantly. The addition of vitamins did not have significant influence on growth of either strains.

Growth rate of the aceticlastic strains was extremely slow, typical for this group of methanogens (explained usually by the lowest energy yield of the aceticlastic methanogenesis), although strain Mx was obviously even more slowed down by the extreme conditions. Since the growth was not exponential, only a rough estimation of the doubling time was possible, and it was around 25–30 d. The molar conversion of acetate to methane was close to the theoretical 1:1 (Supplementary Figure 2A). For strain M04Ac, the specific growth rate in acetate-containing basal medium was 0,011 h^−1^ (doubling time 63 h) (Supplementary Figure 2B). However, it should be taken into account that the enrichment culture grew much faster than the isolate. Upon further purification, growth of strain M04Ac eventually slowed down to 4–5 months, and culture finally became not-transferrable.

**Figure 3:**
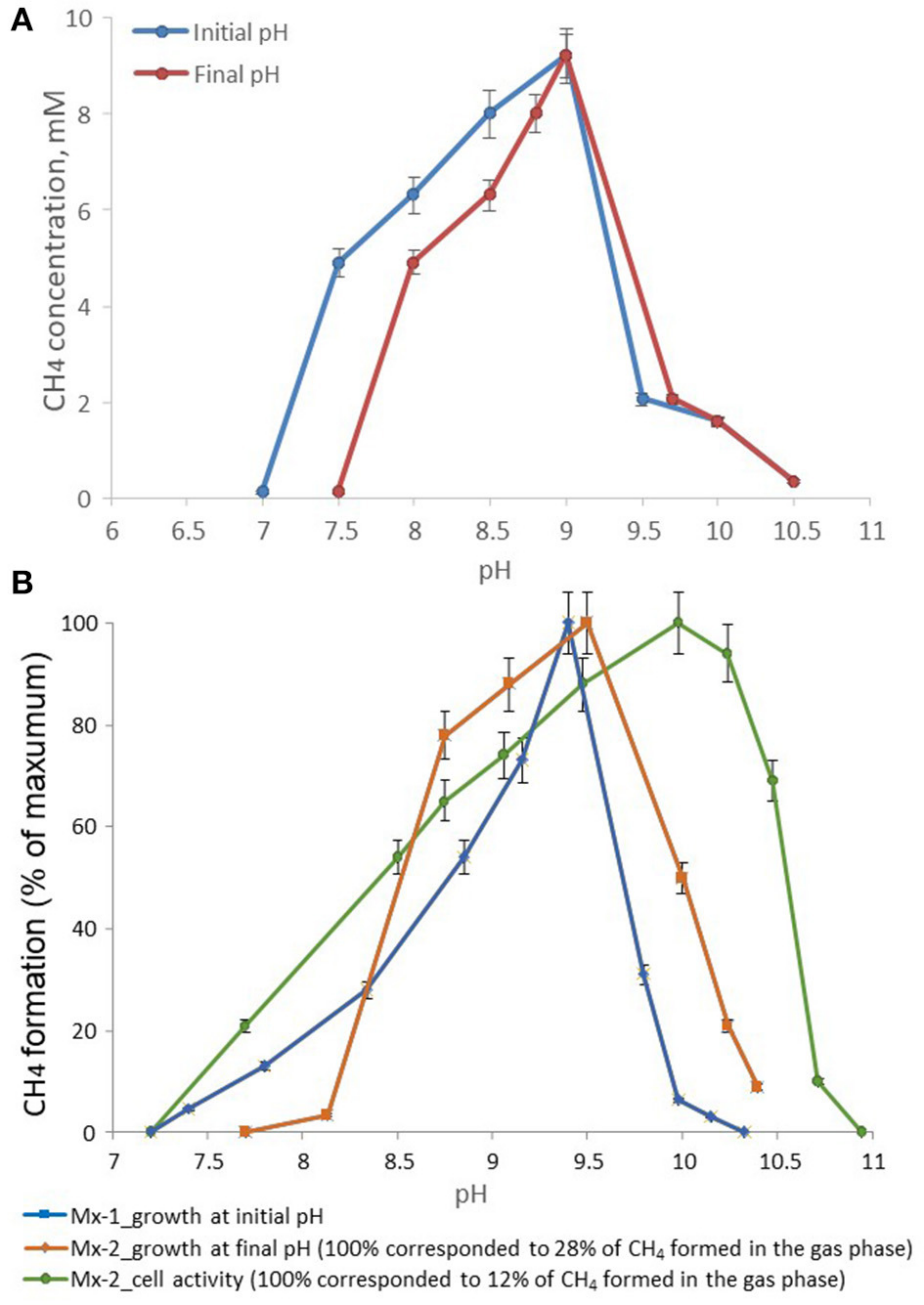
Effect of pH on the methane formation by strains **(A)** M04Ac and **(B)** Mx, cultivated with acetate. The mmol of methane detected in the gas phase were normalized for 1l volume of liquid culture (mM, mmol CH_4_ in the gas phase per liter of liquid culture).

The temperature range for growth of strain M04Ac was 20–45°C, with an optimum at 37°C. No growth was detected at 50°C or above, as well as at 18°C or below after incubation for 4 months. The pH range for growth was 7.5–10.0, with an optimum at pH 9.0. No growth was observed at pH values below 7.0 or above 10.0 (Figure 3A). Thus, strain M04Ac isolated from terrestrial mud volcano was alkalitolerant, similar to the freshwater *Methanotrichaceae* members, and it did not require high sodium concentrations for growth above trace amounts supplied with basic medium. Growth was observed at NaCl concentrations of 0–5.0% (w/v) with an optimum at 0.5–1.0% NaCl (w/v).

**Figure 4:**
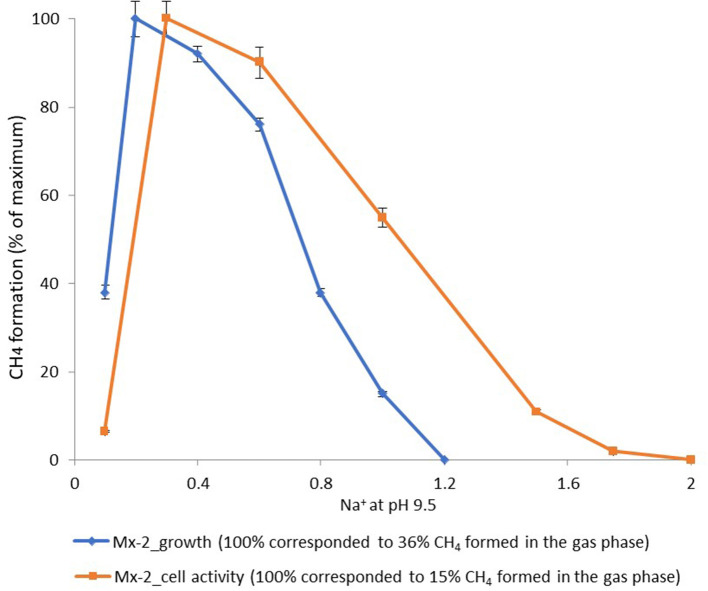
Dependence of the methane formation at pH 9.5 by strain Mx on salt concentration of the medium.

According to the pH-salt profiling, strain Mx represents a moderately salt-tolerant obligate alkaliphile, growing optimally at 0.2–0.3 M total Na^+^ (as sodium carbonates) and within the pH range from 7.8 to 10.2 with an optimum between 9.3 and 9.5. Also typical for the soda lake isolates, the alkalitolerance of methanogenic activity of the Mx resting cells was substantially extended in comparison to the growth optimum in culture (Figures 3B, 4). This means that the organism can still remain metabolically active during periods of elevated alkalinity even without being able to grow.

### Genome statistics

The draft genome of strain M04Ac was assembled into 96 contigs with genome size of 2,444,195 bp and the DNA G + C content of 58.31%. The genome was predicted to contain 2,423 protein-coding sequences and 53 RNA genes, including three complete rRNAs (1 5S, 1 16S, and 1 23S) and 48 tRNAs. The nomenclatural type is GCA_029167205.1^Ts^ according to SeqCode requirements (Hedlund et al., 2022; Whitman et al., 2022). The draft genome of strain Mx was assembled into 100 contigs with genome size of 2,412,901 bp and the DNA G + C content of 58.18%. The genome contained 2,406 protein-coding sequences and 48 RNAs genes, including three complete rRNAs (1 5S, 1 16S, and 1 23S) and 43 tRNAs. Detailed genomes statistics can be found in Supplementary Table 3. The nomenclatural type is GCA_029167045.1^Ts^ according to SeqCode requirements (Hedlund et al., 2022; Whitman et al., 2022). In general, the genomes of M04Ac and Mx strains are similar in size and gene content. We have analyzed the genomic data in relation to methanogenic metabolism and halo-alkaline adaptations of the novel strains.

### Functional genome analysis

#### Cell morphology

Genes distantly similar to the genes encoding bacterial actin homolog MreB, playing an important role in many different cellular processes such as cell shape determination and motility, were found in the genomes of Mx and M04Ac strains (MDF0590229 and MDF0592540, respectively). However, amino acid identities of these putative proteins did not exceed 34% identity depending on the microorganism compared. Other archaeal actin homologs, Arcade and Crenactin, were not encoded in the genomes. Genes, encoding two domains of an actin homolog Ta0583 from euryarchaeon *Thermoplasma acidophilum*, were also absent from the genomes of the strains (Roeben et al., 2006). Several cell division proteins FtsZ, typical for *Euryarchaeota*, were found in both genomes.

The presence of thin long pili (around 10 μm in length) in cells of strain M04Ac was the first evidence for the existence of extracellular appendages within the family *Methanotrichaceae*. However, we failed to detect any genes responsible for its formation. In particular, genes encoding type IVa and IVb pili, as well as Tad pili or type II pseudopili of model bacteria, were absent from the genome of the strain M04Ac (Giltner et al., 2012). Only several putative PilS (MDF0592160; MDF0592073; MDF0594010) and PilU (MDF0593561) homologs encoded in the M04Ac genome demonstrated distant amino acids similarity (around 51.5–62.1%) with corresponding proteins of type IVa pili from *Pseudomonas aeruginosa*, while the other important members of this protein family were not detected in the genome. Likewise, neither type IV pili-like locus characteristic for other methanogens nor multiple structural flagellin genes (*flaABC*) and genes encoding prepilin-like FlaK/PibD-like proteases were found in M04Ac genome (Ng et al., 2008).

#### Acetate transport and activation

Aceticlastic methanogens transport acetate into the cell by a proton symport mechanism using acetate permease (AceP). The AceP homolog is encoded in the genome of *Methanothrix thermophila* (Smith and Ingram-Smith, 2007; Ferry, 2020). We have identified acetate uptake transporter genes homologous to AceP in the genomes of strain M04Ac (MDF0592145) and *Methanothrix harundinacea* 6Ac (AET63818), which have 87% protein sequence identity to each other. The gene of AceP homolog was also identified in strain Mx genome, but this gene is probably impaired, since it encodes only 55 aa compared to 209 aa of AceP protein of strain M04Ac. The AceP homolog genes were localized in a gene cluster similarly organized in strain Mx, strain M04Ac, and *M. harundinacea* 6Ac. This cluster also includes a gene of a γ- carbonic anhydrase (Cam), which is believed to be involved in acetate transport (Ferry, 2020). The Cam genes were rather conserved, having about 80% amino acid identity between the studied strains. In addition to the γ-carbonic anhydrase, the genome of strain M04Ac encoded an extracellular β-carbonic anhydrase (MDF0593542). To the best of our knowledge, the occurrence and function of extracellular β class carbonic anhydrases have not previously been systematically studied in *Archaea*. In the genomes of strain Mx and *M. harundinacea* 6Ac, genes of β-class carbonic anhydrase were not found.

The transported acetate can be converted to acetyl-CoA by the AMP-forming acetyl-CoA synthetase (Acs), which was encoded in the studied genomes in multiple copies (five in strain M04Ac and three in strain Mx). The genomes of both strains did not contain genes of alternative pathway of acetate activation–acetate kinase (Ack) and phosphotransacetylase (Pta). The acetyl-CoA is then decarbonylated by the acetyl-CoA decarbonylase complex (ACDS).

#### C1 branch of the methanogenic pathway

Both genomes encode for seven steps of CO_2_ reduction to methane, namely formylmethanofuran dehydrogenase (*fmdABCE*), formylmethanofuran:tetrahydromethanopterin formyltransferase (*ftr*), methenyltetrahydromethanopterin cyclohydrolase (mch), methylenetetrahydromethanopterin dehydrogenase (*mtd*), methylenetetrahydromethanopterin reductase (*mer*), tetrahydromethanopterin methyltransferase (*mtrABCDEFGH*), and methyl-coenzyme M reductase (*mcrABCDG*) (Supplementary Table 4).

#### Electron transport and energy conservation

The redox reactions in all aceticlastic methanogens include oxidation of reduced ferredoxins and reduction of CoMS-SCoB by two Hdr complexes. Genes encoding soluble F_420_-dependent (Frh) and F_420_-nonreducing (Mvh) hydrogenases, as well as membrane-bound ferredoxin-dependent (Ech) and methanophenazine-dependent (Vho) hydrogenases, common in hydrogenotrophic and methylotrophic methanogens, were absent in genomes of strains Mx and M04Ac. Like the *Methanothrix* species, genomes of strains Mx and M04Ac did not contain genes of H^+^/Na^+^-translocating ferredoxin:NAD oxidoreductase (Rnf complex). Genes of F_420_H_2_:methanophenazine oxidoreductase (Fpo subunits ABCHJNO, lacking FpoF), cytoplasmic (HdrABC), and membrane bound (HdrDE) CoB-CoM heterodisulfide reductases were present in both genomes.

ATP synthase was encoded by a single locus in strain Mx (archaeal A_0_A_1_-type), while M04Ac genome contained two ATP synthase loci, one similar to Mx and another one more resembling the F_0_F_1_ type. Additionally, the genome of strain M04Ac contained *atpZ*/*atpI* gene (MDF0594135), which was located within the operon encoding one of the two ATP synthases. In *Bacillus pseudofirmus* OF4, the product of *atpZ* gene has been shown to increase the ability of this alkaliphile to acquire sufficient magnesium, which is problematic at elevated pH (Hicks et al., 2003). Neither strain Mx nor other representatives of the *Methanotrichaceae* had a similar operon in their genomes. As for the soda lake strain Mx, a possible reason for a specialized system for Mg^2+^ uptake is that Mg^2+^ is poorly soluble in sodium carbonate brines due to the formation of basic magnesium carbonates.

Extracellular cytochrome nanowires, probably responsible for long-range electron transfer in many prokaryotes, have been recently reported (Baquero et al., 2023). However, the genomes of strains Mx and M04Ac did not contain homologs of either PcECN or AvECN. We also did not find homologs of the outer membrane multiheme cytochromes, MtrA, MtrC, OmcS, OmcE, and OmcZ, which have been shown to determine electron transfer in the model bacteria, *Shewanella oneidensis*, and *Geobacter sulfurreducens*.

#### Genomic evidence for halo-alkaline adaptations of strains

Genes encoding multisubunit Na^+^/H^+^ antiporters of the Mrp/Mnh family, normally operating in (halo)alkaliphiles (Ito et al., 2017), were not found in the genomes of either strain. Two monosubunit Na^+^/H^+^ antiporters were encoded in the genome of Mx only (MDF0590085, MDF0591576) but were absent from the M04Ac genome. Both strains possessed multiple cation/proton transporters that exchange K^+^ or Ca^2+^ for external H^+^ (Supplementary Table 5). In particular, the genomes encoded a variety of potassium-trafficking ion pumps including potassium import/export (KefB, TrkA/TrkAH, NhaP). Several symporters of solute/sodium (SSS family) that use an existing sodium gradient to drive the uphill transport of several solutes (amino acids mainly) across the membrane were also found in both genomes (Supplementary Table 5). In particular, both genomes encode several homologs of Na^+^-dependent proline symporter PutP.

Bacterial mechanosensitive channels play a significant role in protecting cells against hypoosmotic shock. Genes encoding the small conductive mechanosensitive ion channel MscS responding both to stretching of the cell membrane and to membrane depolarization were found in both genomes (Supplementary Table 5), while the mechanosensitive channel of large conductance, MscL, was encoded only in the genome of Mx strain (MDF0590401).

Genomic analyses showed that both strains have potential for the biosynthesis of osmoprotector ectoine, which is synthesized from L-aspartate-β-semialdehyde by stepwise transamination (EctB), acetylation (EctA), and closure of the ring (EctC). This pathway is widely distributed in halophilic bacteria but seems to be extremely rare within the archaeal domain (Czech et al., 2018). According to the genomic database, it has been found only in the novel strains together with *M. harundinacea, Mx. soehngenii, Methanobacterium alkalithermotolerans, Methanomicrobia* ANME-1, and ammonia-oxidizing *Thaumarchaeota* of the genus *Nitrosopumilis*. In strain Mx, the genes encoding ectoine biosynthesis enzymes are organized in a single locus (MDF0590640–MDF0590642), while in the M04Ac genome, *ectC* gene is located separately (MDF0592299, MDF0592300, and MDF0594107). However, the complete glycine-betaine/L-proline ABC transporter (*proVWX*, MDF0594108-MDF0594110) is localized near *ectC* in the M04Ac genome. According to the phylogenetic reconstruction performed by Czech et al., representatives of the *Methanotrichaceae* might have obtained ectoine biosynthesis genes by horizontal transfer from *Gammaproteobacteria*.

The genes encoding the ProVWX complex and the BCCT (betaine-choline-carnitine) transporter genes were absent from the genome of the Mx strain. Both genomes lack genes for proline biosynthesis (*proABC*) and trehalose biosynthesis (trehalose-6-phosphate synthase/trehalose-6-phosphatase).

Thus, it appeared that ectoine was the preferred osmo-protectant for these strains. Indeed, a chemical analysis of the strain Mx cells grown at 0.6 M total Na^+^/pH 9.5 confirmed the actual production of ectoine in this methanogen, albeit at a low specific level of approximately 1.5 mg g^−1^ of dry weight (Supplementary Figure 3). The exact value was not possible to determine because the cells did not form a solid pellet upon centrifugation, and the final biomass contained a substantial quantity of salt. It must be stressed that this is the first direct evidence of the ectoine formation in archaea.

## Discussion

Approximately two-thirds of biogenic methane is produced by aceticlastic methanogens, making them major contributors to the global methane budget (Lyu et al., 2018). Members of the genus *Methanothrix* inhabit anaerobic zones of various mesophilic and thermophilic environments, including digesters, marine and freshwater sediments, soils, and thermal springs (Huser et al., 1982; Nozhevnikova and Chudina, 1985; Westermann et al., 1989; Sonne-Hansen and Ahring, 1997; Ma et al., 2006; Mori et al., 2012; Angle et al., 2017; Katayama et al., 2022). Strain Mx represents the first example of aceticlastic methanogen found in saline soda lakes where obligately haloalkaliphilic prokaryotes growing optimally in Na-carbonate brines with pH above nine usually predominate. Aceticlastic methanogenesis might be important for the Kulunda Steppe soda lakes in case of a significant downshift in salinity that regularly occurs in this area (Altai, Russia) or in permanently low-salt soda lakes. Strain M04Ac is isolated from a mud volcano located within the Kerch-Taman mud volcanic province. Many TMVs in this area have pH 8.5–9.1 and contain high concentrations of HCO${\;}_{3}^{-}$ and Na^+^ ions (Lavrushin et al., 2021). The presence of *Methanotrichaceae* species had not previously been detected by 16S rRNA gene-based observations in several TMVs of this region (Merkel et al., 2021; Khomyakova et al., 2022; Slobodkin et al., 2023). However, in the other study, *Methanothrix (“Methanosaeta”) thermophila* accounted for almost 40% of *Archaea* in one TMV of southwestern Taiwan, suggesting the importance of this microbial group in certain TMVs (Cheng et al., 2012).

According to our phylogenomic reconstruction (Figure 1), as well as to the current GTDB classification, strains Mx and M04Ac are clustered together with *Methanothrix harundinacea* within a well separated group of the genus level. All currently characterized *Methanothrix* species are neutrophilic and Na^+^-independent except “*M. pelagicus*,” whose growth requires at least 0.2 M and tolerates up to 0.8 M NaCl (Mori et al., 2012). *M. harundinacea* has a wide pH range up to 9.0, although its pH optimum is typical of that of sodium-independent species (Table 1). Strains Mx and M04Ac are alkaliphilic, they are able to grow at pH 10, and have pH optima at or above 9.0.

**Table 1:** Comparative characteristics of novel strains with the type species of the proposed genus: 1–strain Mx^Ts^ (this study); 2–strain M04Ac^Ts^ (this study); 3–*Methanocrinis harundinaceus* comb. nov. (former *Methanothrix harundinacea, Methanosaeta harundinacea*, Ma et al., 2006*;* Akinyemi et al., 2020).

**Features**	**1**	**2**	**3**
Cell size, length x diameter, μm	1.9–4.8 x 0.6–1.0	1.7–6.5 x 0.9–1.5	3–5 x 0.8–1.0
Cell morphology and motility	Non-motile rod	Non-motile rod, thin pili were found	Non-motile rod
Relation to oxygen	Obligate anaerobe	Obligate anaerobe	Obligate anaerobe
Temperature optimum, ^0^C	28–30	37	34–37
pH optimum	9.3–9.5	9.0	7.2–7.6
Total Na^+^ (M), (optimum)	< 1 (0.2–0.3)	< 0.85 (0.09–0.17)	< 0.2 (0)
Substrate	Acetate	Acetate	Acetate
Doubling time, h	ND^*^	63	28
Compounds required or stimulatory for growth	No dependency	Yeast extract	Peptone, yeast extract
G + C, mol%	58.18	58.31	60.6
Isolation source	Saline soda lake	Terrestrial mud volcano	Anaerobic sludge digester treating beer-manufacture wastewater

* ND, not determined.

The ability of anaerobic alkaliphilic prokaryotes to maintain the pH of the cytoplasm ~2 units below the external pH largely depends on the efficient functioning of electrogenic Na^+^/H^+^ antiporters and on the use of Na^+^-coupled ATPases and the primary sodium pumps creating membrane potential (Banciu and Muntyan, 2015). The ATP synthases in the group of methanogenic archaea are promiscuous and may use Na^+^ and H^+^ simultaneously (Schlegel and Müller, 2013). Although we did not find genes encoding the multisubunit-Na^+^/H^+^ antiporters of the Mrp/Mnh families in the strains Mx and M04Ac, the strains may exploit different strategies for adaptation to the alkaline environment, including single- subunit cation:proton antiporters for the pH homeostasis and synthesis of ectoine for osmoprotection.

Strains Mx and M04Ac are non-motile filamentous rods. In *Archaea*, rod-shaped or filamentous cell morphologies correlate with the presence of Crenactin/Arcadin-1 or actin MreB (Ettema et al., 2011). Still, there are no data on genetic determinants of cytoskeleton in *Methanotrichaceae*.

The mechanisms of interactions of *Methanotrichaceae* with other microorganisms or minerals are poorly understood. We have for the first time detected thin pili/fimbria-like filaments on the cell surface of strain M04Ac using electron microscopy. It might be possible that such filaments facilitate electron transfer between strain M04Ac and its bacterial partners similar to the conversion of ethanol to methane by the co-culture of *Methanothrix harundinacea* and *Geobacter metallireducens* (Rotaru et al., 2014). Yet, in the genome of strain M04Ac we did not find genes of PilA (e-pilin) or other pilin-encoding genes, which suggests that the observed belong to a so far unknown type of archaeal filament. This prompts reconsideration of the possible role of *Methanotrichaceae* species in syntrophic interactions.

Overall, this work demonstrates for the first time that the low energy aceticlastic methanogenesis might still be active at unfavorable conditions present in (halo) alkaline habitats.

Based on the distinct phylogenetic position within the family *Methanotrichaceae* (Figure 1 and GTDB), we propose to reclassify *Methanothrix harundinacea* as the type species of a new genus *Methanocrinis* gen. nov., as *Methanocrinis harundinaceus* sp. nov., comb. nov. According to phylogenetic position and phenotypic, physiological, and genomic properties of strains Mx and M04Ac, we propose under SeqCode the complete genome sequences of strain Mx^Ts^ (GCA_029167045.1) and strain M04Ac^Ts^ (GCA_029167205.1) as nomenclatural types of *Methanocrinis natronophilus* sp. nov. and *Methanocrinis alkalitolerans* sp. nov., respectively, which represent two other species within this new genus.

## Description of *Methanocrinis* gen. nov.

*Methanocrinis* (Me.tha.no.cri'nis. N.L. neut. n. *methanum*, methane; L. masc. n. *crinis*, hair; N.L. masc. n. *Methanocrinis*, methane (-producing) hair).

Straight, rod-shaped cells with flat ends, non-motile. Organotrophic, obligate aceticlastic methanogens converting acetate into methane and CO_2_. Represented by neutrophilic and alkaliphilic species. Separation of the genus is justified by its distinct genome-based phylogenetic position.

The type species is *Methanocrinis harundinaceus*.

## Description of *Methanocrinis harundinaceus* comb. nov.

*Methanocrinis harundinaceus* (ha.run.di.na'ce.us. L. masc. adj. *harundinaceus*, like a reed, referring to the cell shape of a reed stem).

The description of the species is identical to that given for *Methanosaeta harundinacea* (Ma et al., 2006). Assignment of the species into a new genus is justified by its distinct phylogenetic position within a concatenated protein gene tree within the family *Methanotrichaceae*.

Synonyms: *Methanosaeta harundinacea* (Ma et al., 2006), *Methanothrix harundinacea* (Akinyemi et al., 2020).

## Description of *Methanocrinisalkalitolerans*^Ts^

*Methanocrinis alkalitolerans* (al.ka.li.to'le.rans. N.L. neut. n. *alkali*, from Arabic n. (*al-qaliy*), the soda ashes; L. part. adj. *tolerans*, tolerating; N.L. part. adj. *alkalitolerans*, tolerating high alkalinity).

Cells are non-motile, rod-shaped, 1.7–6.5 μm in length, and 0.9–1.5 μm in diameter. Can form polar pili/fimbriae-like structures of unknown nature on the surface of the cell. Filaments are formed after long incubation times. Growth occurs at 20–45°C (optimum, 37°C) and at pH 7.5–10.0 (optimum 9.0); the presence of NaCl is not required. Yeast extract is not essential for growth but highly stimulatory. Utilizes acetate for methane production. No growth or CH_4_ formation is observed on H_2_/CO_2_, formate, carbon monoxide, and methanol. The complete genome of strain M04Ac^Ts^, available under the GenBank assembly accession number (GCA_029167205^Ts^), is the designated nomenclatural type for the species and was recovered from an enrichment culture, cultivated on acetate, and established from a terrestrial mud volcano at the Taman Peninsula, Russian Federation. The genome is characterized by a size of 2.44 Mb and a G + C content of 58.31 mol%. Completeness is estimated by CheckM at 99.84% with 0.00% contamination. The GenBank accession number for the 16S rRNA gene sequence of M04Ac^Ts^ is OQ918309.

## Description of *Methanocrinisnatronophilus*^Ts^

*Methanocrinis natronophilus* [na.tro.no.phi'lus. N.L. n. *natron* (arbitrarily derived from the Arabic n. *natrun* or *natron*), soda; N.L. pref. *natrono*-, pertaining to soda; N.L. adj. *philus* (from Gr. fem. adj. *phile*), friend, loving; N.L. adj. *natronophilum* soda-loving].

Cells are non-motile, rod-shaped, 1.9–4.8 x 0.6–1.0 μm. Forms multicellular filaments in a common sheath. Forms methane exclusively from acetate by the aceticlastic pathway. Obligately alkaliphilic with the pH range for growth from 7.5–7.8 to 10.2 (optimum at 9.3–9.5). NaCl is not required for growth, but up to 1 M total Na^+^ in the form of sodium carbonates is tolerated. The non-growing cells still actively produce methane at pH up to 10.5 and 1.5 M total Na^+^. Ammonium serves as the nitrogen source. Optimal growth temperature is 35°C. Yeast extract is not essential for growth but slightly stimulatory. The complete genome of strain Mx^Ts^, available under the GenBank assembly accession number (GCA_029167045^Ts^) is the designated nomenclatural type for the species and was recovered from an enrichment culture, cultivated on acetate, and established from a saline soda lake, in southwestern Siberia, Russia. The genome of the type strain is 2.41 Mb with the G + C content of 58.18 mol%. Completeness is estimated by CheckM at 97.04% with 0.00% contamination. The GenBank accession number for the 16S rRNA gene sequence of Mx^Ts^ is KP205578.

## Data availability statement

The datasets presented in this study can be found in online repositories. The names of the repository/repositories and accession number(s) can be found in the article/Supplementary material.

## Author contributions

DS and MK conducted the research. DS, AS, MK, and AM wrote the manuscript. DS was involved in funding acquisition. All authors contributed to the article and approved the submitted version.
