# Supplementary material for: Phenotypic and genomic characterization of the first alkaliphilic aceticlastic methanogens and proposal of a novel genus Methanocrinis gen.nov. within the family Methanotrichaceae
Source: Front Microbiol. 2023 Oct 11;14:1233691. doi: 10.3389/fmicb.2023.1233691 (PMC10598746; doi:10.3389/fmicb.2023.1233691)
Supplement: Supplementary file 1 [file Data_Sheet_1.PDF]

Phenotypic and genomic characterization of the first alkaliphilic aceticlastic methanogens and proposal of a novel genus *Methanocrinis* gen. nov. within the family *Methanotrichaceae*

Maria A. Khomyakova[1*], Alexander Y. Merkel[1], Alexander I. Slobodkin[1] and Dimitry Y. Sorokin[1,2*]

*** Correspondence:** M.A. Khomyakova mary_klimova@mail.ru

D.Y. Sorokin d.sorokin@tudelft.nl

SUPPLEMENTARY MATERIALS

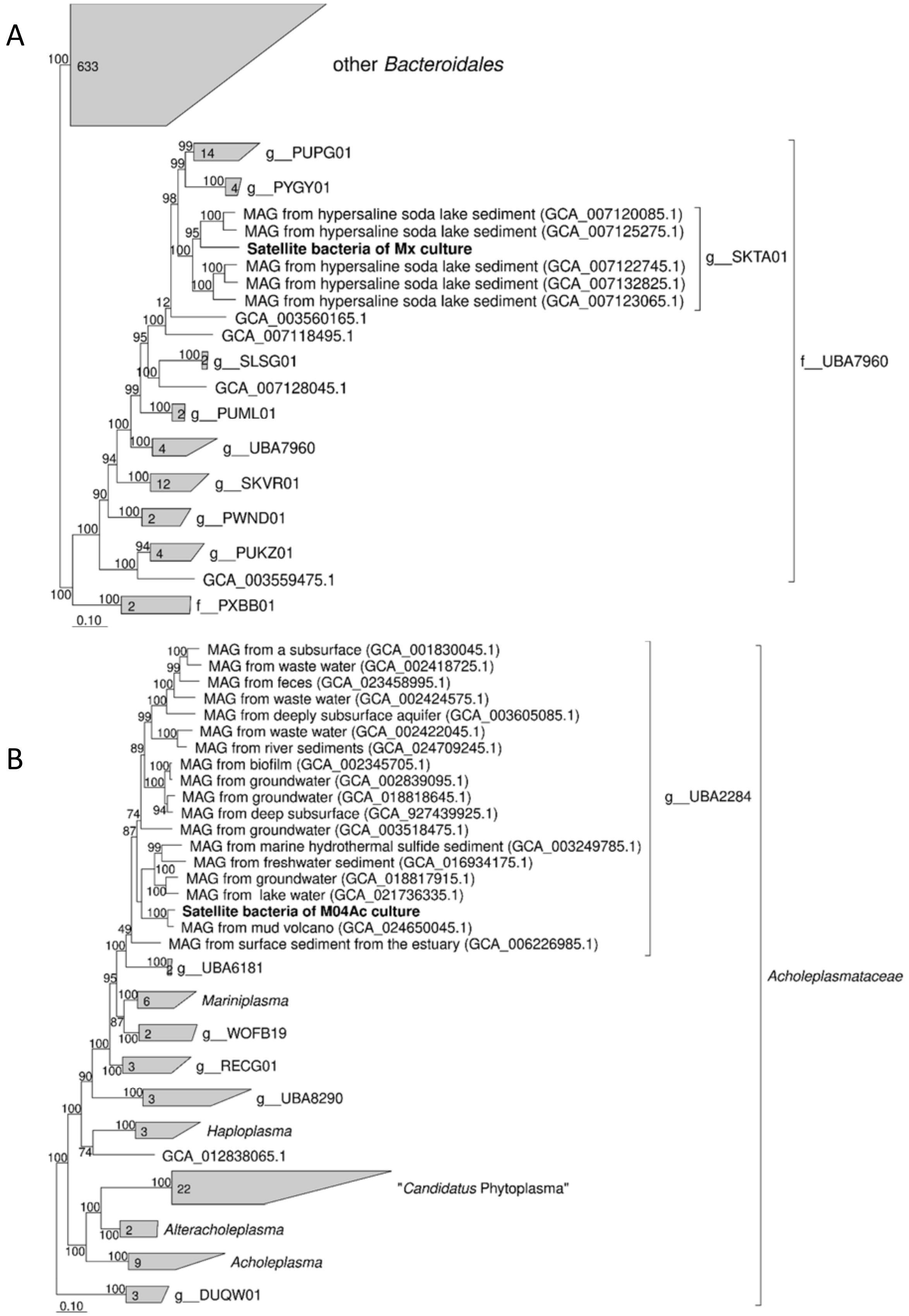

Supplementary figure S1. Phylogenomic analysis of satellite bacteria of (A) Mx culture and (B) M04Ac culture based on 120 conserved single-copy bacterial protein-coding marker genes conducted using GTDB-Tk v2.3.0 de_novo workflow (Chaumeil et al., 2022).

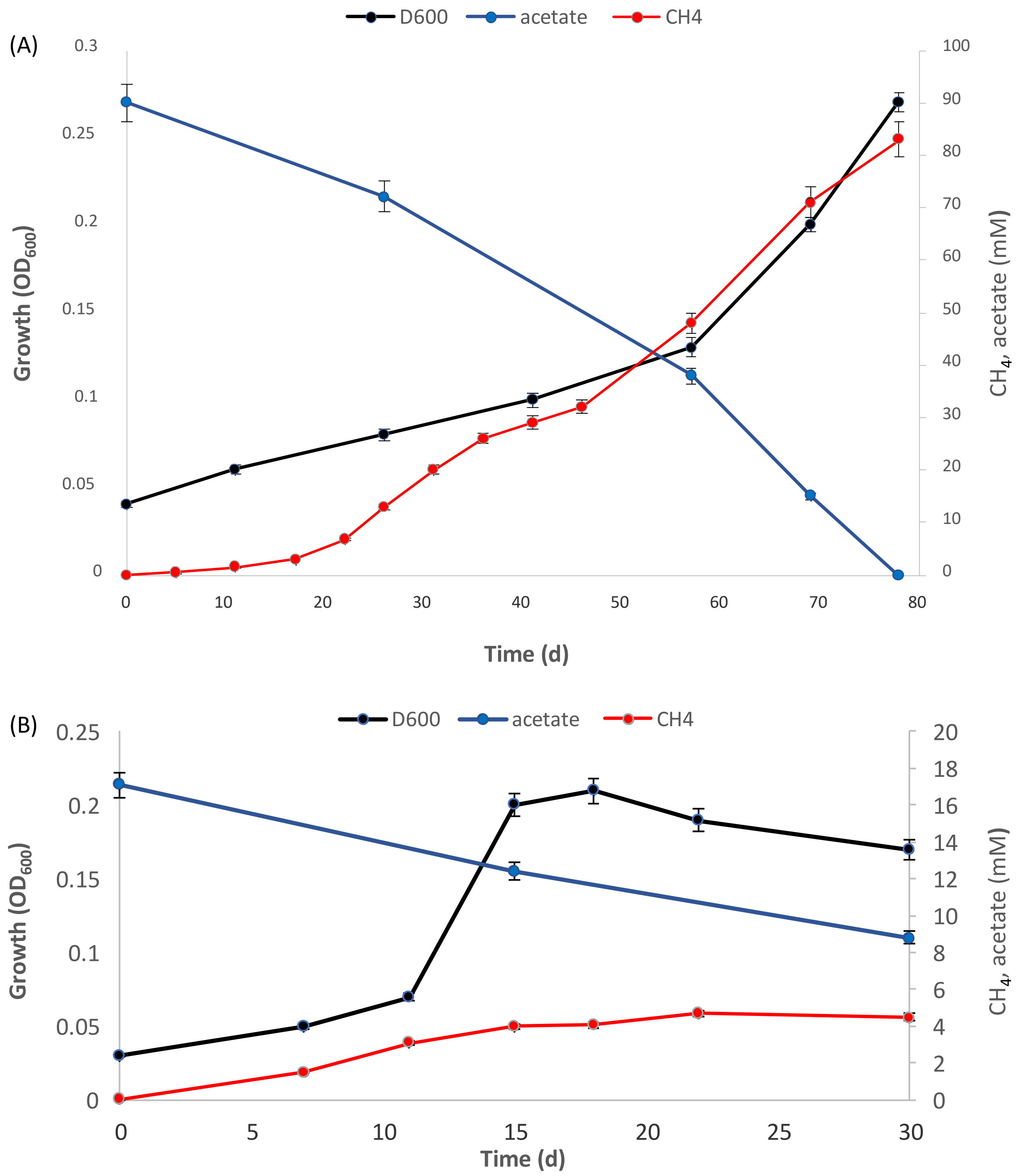

Supplementary figure S2. Growth dynamics of the strains (A) Mx and (B) M04Ac cultivated with acetate. The mmol of methane detected in the gas phase were normalized for 1l volume of liquid culture, (mM = mmol CH_4 in the gas phase per liter of liquid culture).

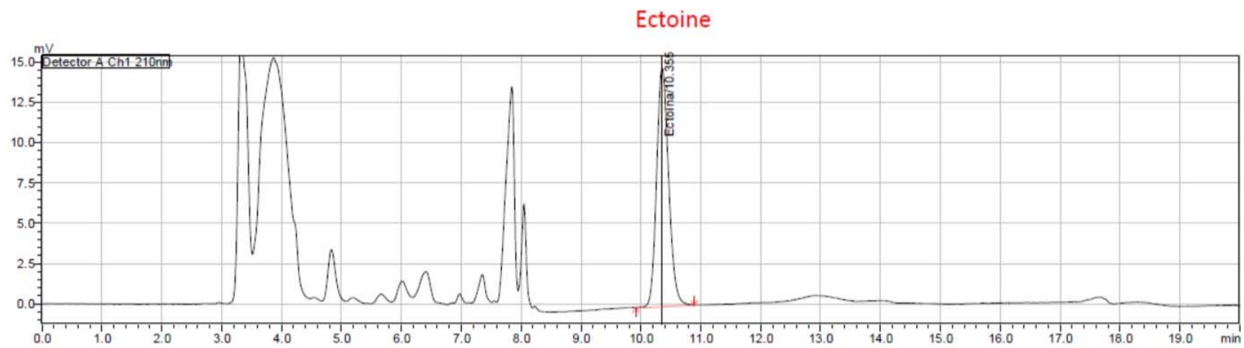

Supplementary figure S3. HPLC chromatogram showing a presence of ectoine in the cells of *Methanocrinis* strain Mx grown with acetate in a sodium carbonate buffer containing 0.6 M total Na^{+} at pH 9.5.

Supplementary Table 1. 16S rRNA and *mcrA* gene sequences identities, ANI and AAI values between strain Mx, strain M04Ac, *M. harundinacea*, *M. thermoacetophila* and *M. soehngenii*.

	strain Mx	strain M04Ac	*M. harundinacea*	*M. thermoacetophila*	*M. soehngenii*
16S rRNA gene sequences identity (%)					
strain Mx	-	98.03	98.64	92.29	91.54
strain M04Ac	98.03	-	98.30	92.27	91.60
M. harundinacea 6Ac (CP003117)	98.64	98.30	-	92.55	91.62
M. thermoacetophila DSM 4774 (LN868388)	92.29	92.27	92.55	-	91.95
M. soehngenii (X51423)	91.54	91.60	91.62	91.95	-
mcrA gene AA sequences identity (%)					
strain Mx (MDF0590866)	-	95.53	88.73	84.79	83.01
strain M04Ac (MDF0592261)	95.53	-	88.37	85.33	84.44
M. harundinacea 6Ac (AET63880)	88.73	88.37	-	86.23	82.47
M. thermoacetophila DSM 4774 (ABK14360)	84.79	85.33	86.23	-	88.55
M. soehngenii (AEB67565)	83.01	84.44	82.47	88.55	-
ANI (%)					
strain Mx (GCA_029167045.1)	-	87.30	85.65	85.90	81.86
strain M04Ac (GCA_029167205.1)	87.30	-	85.70	86.74	81.29
M. harundinacea 6Ac (GCF_000235565.1)	85.65	85.70	-	85.00	83.19
M. thermoacetophila DSM 4774 (GCF_000014945.1)	85.90	86.74	85.00	-	81.29
M. soehngenii (GCF_000204415.1)	81.86	81.29	83.19	81.29	-
AAI (%)					
strain Mx (GCA_029167045.1)	-	83.72	79.21	62.97	62.56
strain M04Ac (GCA_029167205.1)	83.72	-	80.18	63.28	62.93
M. harundinacea 6Ac (GCF_000235565.1)	79.21	80.18	-	63.21	62.65
M. thermoacetophila DSM 4774 (GCF_000014945.1)	62.97	63.28	63.21	-	63.84
M. soehngenii (GCF_000204415.1)	62.56	62.93	62.65	63.84	-

Supplementary Table 3. Genome properties of the two strains of the genus *Methanocrinis* gen. nov.. comb. nov.

Attributes	Strain M04Ac	Strain Mx
Sequence size (bp)	2.444.195	2.412.901
Number of contigs	96	100
GC content (%)	58.31	58.18
Longest contig size	128.298	107.349
N50 value	58.935	45.186
L50 value	15	18
Total number of genes	2.499	2.490
Total CDSc	2.446	2.442
Pseudogenes	23	36
Number of RNAs (tRNAs. rRNAs. ncRNAs)	53 (48. 3. 2)	48 (43. 3. 2)
Completeness. %*	99.84	97.04
Contamination. %*	0.00	0.00
Genbank Accession number	JARFPL000000000	JARFPK000000000

* Analysed by CheckM v1.0.12 lineage_wf (Parks et al.. 2015).

Supplementary Table 4. Genes of the methanogenic pathway in strains.

Step of methano-genesis	Protein name	Encoding gene	Strain Mx Locus tag	Strain M04Ac Locus tag
1	Methyl-coenzyme M reductase subunit A	*mcrA*	MDF0590866.1	MDF0592261.1
	Subunit B	*mcrB*	MDF0590869.1	MDF0592258.1
	Subunit C	*mcrC*	MDF0590747.1	MDF0592601.1
	Subunit D	*mcrD*	MDF0590868.1	MDF0592259.1
	Subunit G	*mcrG*	MDF0590867.1	MDF0592260.1
2	tetrahydromethanopterin S-methyltransferase subunit A	*mtrA*	MDF0589847.1	MDF0593573.1
	tetrahydromethanopterin S-methyltransferase subunit A (2)	*mtrA*	MDF0591194.1	MDF0593933.1
	Subunit B	*mtrB*	MDF0591195.1	MDF0593934.1
	Subunit C	*mtrC*	MDF0591196.1	MDF0593935.1
	Subunit D	*mtrD*	MDF0591197.1	MDF0593936.1
	Subunit E	*mtrE*	MDF0591198.1	MDF0593937.1
	Subunit F	*mtrF*	MDF0591193.1	MDF0593932.1
	Subunit G	*mtrG*	MDF0591192.1	MDF0593931.1
	Subunit H	*mtrH*	MDF0591191.1	MDF0593930.1
	Subunit H (2)	*mtrH*	MDF0591753.1	MDF0594143.1
3	Coenzyme F420-dependent N5N10-methylene tetrahydromethanopterin reductase	*mer*	MDF0591619.1	MDF0592046.1
4	Methylenetetrahydromethanopterin dehydrogenase	*mtd*	MDF0591750.1	MDF0593919.1
5	Methenyltetrahydromethanopterin cyclohydrolase	*mch*	MDF0590263.1	MDF0594041.1
6	Formylmethanofurantetrahydromethanopterin N-formyltransferase	*ftr*	MDF0591597.1	MDF0592499.1
7	Formylmethanofuran dehydrogenase subunit A	*fmdA*	MDF0589827.1	MDF0593190.1
	Subunit B	*fmdB*	MDF0589830.1	MDF0593189.1
	Subunit C	*fmdC*	MDF0589828.1	MDF0593191.1
	Subunit E	*fmdE*	MDF0589622.1	MDF0593012.1
	Formylmethanofuran dehydrogenase subunit A (2)	*fmdA*	MDF0589911.1	MDF0593641.1
	Subunit B (2)	*fmdB*	MDF0591332.1	MDF0593638.1
	Subunit C (2)	*fmdC*	MDF0589912.1	MDF0593640.1
	Subunit E (2)	*fmdE*	MDF0590190.1	-
	Subunit B (3)	*fmdB*	MDF0591332.1	MDF0593324.1

Supplementary Table 5. Halo-alkaline adaptation in the strains.

Protein name	Predicted function	Mx Locus tag	M04Ac Locus tag
Membrane secondary ion pumps			
GerN/CPA1	Na^+/K^+:proton antiporter	MDF0590085.1	ND
NhaP/CPA2	Na^+/K^+:proton antiporter	MDF0591576.1	ND
KefB	K^+/H^+: antiporter	MDF0589840.1	MDF0593626.1
TrkH	K^+: H^+ symporter	MDF0589972.1	MDF0592685.1
TrkA(1)	K^+: H^+ symporter	MDF0589972.1	MDF0592685.1
TrkA(2)	K^+: H^+ symporter	MDF0589972.1	MDF0592685.1
MaX1	Ca^{2+}/Na^+ antiporter (archaeal type)	MDF0591278.1	MDF0594082.1
CaCA	Ca^{2+}/Na^+ antiporter	MDF0591866.1	MDF0592267.1
Sodium-dependent symporters			
	Na^+:amino acid symporter	MDF0590146.1	MDF0593945.1
	Na^+:aminoacids symporter (neurotransmitter family)	MDF0590637.1	MDF0592305.1
SSS family	Na^+:solute symporter	MDF0591450.1	MDF0593346.1
PutP	Na^+:proline symporter	MDF0590398.1	MDF0593496.1
PutP	Na^+:proline symporter	MDF0591858.1	MDF0594019.1
Haloadaptation complexes			
MscS	small conductive mechanosensitive ion channel (hypoosmotic protection)	MDF0589841.1	MDF0592713.1
MscL	large conductive mechanosensitive ion channel (hypoosmotic protection)	MDF0590401.1	ND
ProV	glycine betaine/L-proline ABC transporter ATP-binding protein	ND	MDF0594108.1
ProW	proline/glycine betaine ABC transporter permease	ND	MDF0594109.1
ProX	glycine betaine ABC transporter substrate-binding protein	ND	MDF0594110.1
EctC	ectoine synthase EctC (biosynthesis of osmoprotectant ectoine)	MDF0590640.1	MDF0594107.1
EctB	diaminobutyrate-2-oxoglutarate transaminase (ectoine formation)	MDF0590641.1	MDF0592300.1
EctA	diaminobutyrate acetyltransferase (ectoine formation)	MDF0590642.1	MDF0592299.1

REFERENCES

Ma, K., Liu, X., and Dong, X. (2006). *Methanosaeta harundinacea* sp. nov.. a novel acetate-scavenging methanogen isolated from a UASB reactor. Int. J. Syst. Evol. Microbiol. 56. 127–131. doi: 10.1099/ijs.0.63887-0

Parks, D. H., Imelfort, M., Skennerton, C. T., Hugenholtz, P., and Tyson, G. W. (2015). CheckM: assessing the quality of microbial genomes recovered from isolates. single cells. and metagenomes. Genome res. 25. 1043–1055. doi: 10.1101/gr.186072.114

Chaumeil, P. A., Mussig, A. J., Hugenholtz, P., and Parks, D. H. (2022). GTDB-Tk v2: memory friendly classification with the genome taxonomy database. Bioinformatics (Oxford. England). 38(23). 5315–5316. https://doi.org/10.1093/bioinformatics/btac672
